# HIV Self-testing and the Missing Linkage

**DOI:** 10.1371/journal.pmed.1001101

**Published:** 2011-10-04

**Authors:** Rochelle P. Walensky, Ingrid V. Bassett

**Affiliations:** 1 Division of Infectious Disease, Department of Medicine, Massachusetts General Hospital, Boston, Massachusetts, United States of America; 2 Division of General Medicine, Department of Medicine, Massachusetts General Hospital, Boston, Massachusetts, United States of America; 3 Division of Infectious Disease, Department of Medicine, Brigham and Women's Hospital, Boston, Massachusetts, United States of America; 4 Center for AIDS Research, Boston, Massachusetts, United States of America; PLoS Medicine, United Kingdom

## Abstract

Rochelle Walensky and Ingrid Bassett discuss new research in <I>PLoS Medicine</I> that assessed the feasibility of home-based oral HIV self-testing in Malawi, and suggest that linkage to care must be demonstrated before the success of oral self-testing can be determined.

**Linked Research Article**

This Perspective discusses the following new study published in *PLoS Medicine*:

Choko AT, Desmond N, Webb EL, Chavula K, Napierala-Mavedzenge S, et al. (2011) The Uptake and Accuracy of Oral Kits for HIV Self-Testing in High HIV Prevalence Setting: A Cross-Sectional Feasibility Study in Blantyre, Malawi. PLoS Med 8(10): e1001102. doi:10.1371/journal.pmed.1001102

Augustine Choko and colleagues assess the uptake and acceptability of home-based supervised oral HIV self-testing in Malawi, demonstrating the feasibility of this approach in a high-prevalence, low-income environment.

Whatever the next hottest, scientifically proven HIV treatment or prevention strategies are, they will share a common denominator for implementation: the HIV test. Whether accessing preexposure prophylaxis (PrEP), pursuing treatment as prevention (“test and treat”), or taking ART to improve individual clinical outcomes, each begins with learning one's HIV status. With HIV testing as the necessary gateway to so many interventions, the paucity of people in resource-limited settings who have ever had an HIV test is astonishing. Despite over 100,000 facilities in low- and middle-income countries with HIV testing and counseling capacity, only 34% of women and 17% of men have ever had an HIV test in these settings [1]. These facts call for novel and far-reaching approaches to HIV screening, and self-testing is one of them.

In this issue of *PLoS Medicine,* Augustine Choko and colleagues are among the first to describe the feasibility of an HIV self-testing approach [2]. The paper demonstrated the possible: randomly chosen participants in high-density suburbs of Blantyre, Malawi given modest direction could, and did, accurately conduct oral HIV tests and required minimal supervision. Only 8% of subjects chose not to test, and self-testing results were 99% concordant with rapid finger-stick tests collected in parallel. Even more promising, nearly half of the participants were men, a demographic population that has been notoriously hard to engage in testing initiatives in resource-limited settings [1],[3].

## Promise and Potential Obstacles to Widespread Self-testing Implementation

Despite Choko and colleagues' encouraging results and the potential to reach an untapped population, excitement about implementation of self-testing must be tempered. Over half of the population sampled in this study report previous HIV testing, and nearly a quarter had tested within the past year. Demonstrating acceptability of self-testing in those with a prior testing history is only the first step towards ensuring that self-testing is generally feasible. Demonstrating its impact in less-engaged populations, such as those who refused or were never previously offered testing, remains to be seen. Self-testing has been available in the US for over a decade; its use there—where clients must be motivated enough to pay for it themselves—has had minimal impact on diminishing the large population of those needing testing. Untested people have generally lacked initiative and/or finances to engage in self-testing, and 12% of those identified as infected through self-testing were those disbelieving and confirming a prior positive test [4]. While the HIV prevalence and economics differ vastly between the US and Malawi, self-testing will still require individual initiative yet to be demonstrated beyond the study setting.

Through increased convenience, decreased stigma, and heightened privacy, that self-testing might improve testing acceptability seems obvious. Even so, the establishment of any new testing mechanism generally faces unforeseen obstacles. In the case of self-testing, these challenges may include: demonstration of adequate participant buy-in; availability of an easy-to-use and accurate test that is durable to field conditions; sufficient understanding by participants to properly conduct the test and obtain the correct result; and timely access to a health care system equipped to answer questions, assess for, and provide necessary treatment. Shortfalls in any one of these areas may result in a testing program that not only fails, but does harm. Look no further than the 2008 experience in Lesotho—a country with a soaring HIV prevalence of 23% and in dire need of increased stigma-free HIV case identification—to see that a poorly conceived and executed testing program can backfire toward an infringement on human rights and a campaign away from further testing efforts [5].

## Linkage to Care as a Measure of Testing Success

One vital consideration as self-testing traverses from the feasible to the implemented is the issue of linkage to care. The completion of a testing program—its effective successful “endpoint” of diagnosing a previously unidentified case—is not only documenting that the patient received the appropriate result, but also that the positive result ignited a cascade of events leading to timely and effective access to HIV-related care. Even in highly successful clinic- or facility-based HIV testing sites with accessible ART programs, poor uptake of CD4 count testing and subsequent assessment for ART has been documented throughout sub-Saharan Africa [6]–[8]. A systematic review of retention in care between testing and treatment in sub-Saharan Africa estimates that less than 20% of tested patients completed all the necessary steps in the care cascade [9]. If self-testing is preferred by people who feel healthy and are motivated by privacy and an unwillingness to acknowledge their HIV status to others, those who self-test may be even more likely to delay access to care compared to people feeling ill undergoing HIV testing in a health care facility. Even if this is not the case, linkage to care for those testing in locations outside the health care system is likely to be a challenge. As self-testing programs are designed and implemented, they must provide convenient, free, and easily accessible sites of referral—accommodating those most in need of proximity as well as those who strive for the privacy that distance allows. Beyond making care accessible, the next phase of self-testing feasibility studies must evaluate the completion of the care cascade from testing to treatment to demonstrate true self-testing success.

Given severe limitations in prevention and treatment funding, such resources must be wisely invested. Cost-effectiveness studies reported that HIV screening in resource-limited settings represents a worthy investment [10]. Such studies, however, also demonstrated that for equal efficacy of an intervention in the testing pathway, investments are best targeted at later stages in the testing and care cascade—and most efficiently at interventions to promote linkage to care [11],[12]. That is, to ensure investments are well-targeted in the scale up of self-testing programs, linkage to care is a critical evaluation measure. How to conduct those linkage-to-care studies, while maintaining the privacy that self-testing demands, will be among the next phase of self-testing implementation challenges.

## Author Contributions

Wrote the first draft of the manuscript: RPW IVB. Contributed to the writing of the manuscript: RPW IVB. ICMJE criteria for authorship read and met: RPW IVB. Agree with manuscript's results and conclusions: RPW IVB.
